# Bioinformatics and experimental validation identify biomarkers for diagnosing Alzheimer’s disease

**DOI:** 10.3389/fnagi.2025.1566929

**Published:** 2025-08-06

**Authors:** Hui Liu, Chenye Li, Congchen Zhai, Mei Li, Lan Ma

**Affiliations:** ^1^The Second Affiliated Hospital of Harbin Medical University, Harbin, China; ^2^Xi'an North Hospital, Xi'an, China; ^3^Tangdu Hospital of Air Force Medical University, Xi'an, China

**Keywords:** Alzheimer’s disease, bioinformatics, c-Myc, biomarkers, ELISA

## Abstract

**Background and purpose:**

Alzheimer’s disease (AD) is a complex condition involving multiple mechanisms, primarily characterized by the progressive decline in cognition and memory. At present, there is no simple and reliable diagnostic method available for clinical application. Therefore, this study aims to identify potential biomarkers for AD using bioinformatics, providing new insights into its diagnosis.

**Methods:**

This study utilized the transcriptome dataset GSE63060 from the Gene Expression Omnibus (GEO) and applied bioinformatics approaches to identify candidate genes. Differentially expressed genes (DEGs), weighted gene co-expression network analysis (WGCNA), protein–protein interaction (PPI) networks, and machine learning techniques (LASSO, SVM-RFE, Boruta, and XGBoost) were employed on the GSE63060 dataset. Subsequently, the expression levels of the candidate genes were evaluated, and a receiver operating characteristic (ROC) curve was constructed to identify hub genes and establish a corresponding network. Finally, we focused on the common upstream transcription factor c-Myc among the hub genes and conducted clinical experiments to validate its potential. Serum samples were collected from 41 AD patients treated at the Second Affiliated Hospital of Harbin Medical University between October 2023 and November 2024, along with 41 control subjects. The c-Myc protein concentration was measured using ELISA, and a ROC curve was constructed to assess its diagnostic potential.

**Results:**

This study identified four hub genes associated with AD: RPL36AL, NDUFA1, NDUFS5, and RPS25. Additionally, the concentration of the c-Myc protein was significantly different between the AD and control groups (*p* < 0.001). The diagnostic sensitivity was 87.8%, specificity was 51.2%, and the area under the curve (AUC) value was 0.753, suggesting that c-Myc has independent diagnostic significance for AD.

**Conclusion:**

Our study demonstrates that RPL36AL, NDUFA1, NDUFS5, and RPS25 have potential as biomarkers for the diagnosis of AD. Additionally, the experiment suggests that c-Myc could serve as a promising blood biomarker for the diagnosis of AD.

## 1 Introduction

Alzheimer’s disease (AD) is a disorder characterized by a progressive decline in cognitive function, with an insidious onset and an unclear pathogenesis (Florian et al., 2023). Currently, 44 million people worldwide are affected by dementia. This number is projected to more than triple by 2050 as the global population ages, and the annual cost of dementia in the United States alone could exceed $600 billion (Lane et al., 2018). Extracellular *β*-amyloid protein (Aβ) deposition, microtubule-associated protein tau (MAPT) phosphorylation, and neuronal loss are considered key pathological changes in AD (Lucey et al., 2023). AD has become a major health challenge, impacting the quality of life of the elderly and the well-being of their families. Therefore, early identification and intervention are crucial for accurately assessing an individual’s cognitive status and brain health, ultimately improving patients’ quality of life.

AD is a continuum that encompasses preclinical AD, AD-related mild cognitive impairment, and AD-related dementia. The diagnostic process for AD is complex and costly, primarily relying on cerebrospinal fluid analysis, positron emission tomography (PET), and blood biomarker detection. The specificity of Aβ42/40 in cerebrospinal fluid testing ranges from 72 to 89%, but it is an invasive procedure that is often not well accepted by patients, limiting its clinical applicability. Aβ-PET has a specificity of approximately 81 to 93%, with a positive result confirming the presence of Aβ. However, a negative result can essentially rule out AD. Despite its high diagnostic accuracy, Aβ-PET is not widely utilized due to its prohibitive cost (Harrison et al., 2023). Identifying a diagnostic method for AD that is both minimally invasive and cost-effective for clinical practice is a critical issue that requires attention in the current study. Blood biomarker detection is increasingly viewed as a convenient, economical, and non-invasive method, but its specificity remains limited. For example, the specificity of plasma Aβ42/40 ranges from 65 to 78%. Furthermore, integrating multiomics-based biomarkers, including metabolites, lipids, cholesterol biosynthesis, purine metabolism, lipoproteins, bile acids, and genetics, along with their relationship to pathological amyloid and tau networks, could improve the sensitivity of AD diagnosis. This approach may also reveal diverse and complementary molecular pathways that contribute to the early diagnosis and prevention of AD (Zhou, 2021).

Through bioinformatics, this study identifies biomarkers with high specificity, offering valuable insights for the specific diagnosis of AD, as well as for clinical trials, cellular studies, and animal models (Matsuoka and Yashiro, 2024). This study aims to perform bioinformatics analysis of peripheral blood gene expression data from AD patients in the Gene Expression Omnibus (GEO) database to identify potential hub genes, including RPL36AL, NDUFA1, NDUFS5, and RPS25. Additionally, due to limitations of the kit, clinical trials investigating c-Myc, a common transcription factor upstream of the hub genes, are conducted to explore new directions for potential biomarkers of AD.

## 2 Materials and methods

### 2.1 Data acquisition and processing

The GEO^1^ is a high-throughput sequencing repository provided by the National Center for Biotechnology Information. It integrates a vast array of chip and next-generation sequencing data contributed by research institutions worldwide and is freely accessible to researchers. This study utilizes two AD datasets from GEO: GSE63060 and GSE63061 (Sood et al., 2015). GSE63060, based on the GPL10904 platform, includes peripheral blood gene expression profiles from 145 AD patients and 104 healthy controls. GSE63061, based on the GPL10558 platform, includes peripheral blood gene expression profiles from 139 AD patients and 109 healthy controls. In this study, GSE63060 is used as the training set, and GSE63061 serves as the validation set. R software is employed to process the raw data, normalize the dataset, and annotate gene names.

### 2.2 Acquisition of differentially expressed genes (DEGs)

The identification of DEGs in this study was performed using the ‘limma’ package on the R platform to analyze the GSE63060 dataset and identify DEGs between AD patients and healthy individuals. Visualization was conducted using the ‘ggplot2’ package in R to generate volcano plots. DEGs were selected based on the criteria of |log2 fold change| > 0.585 and *p*-value < 0.05 (Wang et al., 2024).

### 2.3 The weighted gene co-expression network analysis (WGCNA)

The ‘WGCNA’ package in R was used to analyze the GSE63060 dataset, grouping genes with similar or identical co-expression patterns into modules (Langfelder and Horvath, 2008). The module most strongly associated with AD was identified as the key WGCNA module based on Pearson correlation.

### 2.4 Acquisition of intersection genes

The ‘venn.diagram’ function in R was used to obtain the intersection of the key WGCNA modules and DEGs, respectively, in order to identify the common genes related to AD across the two datasets.

### 2.5 Construct protein-protein interaction (PPI) network

Firstly, the STRING database^2^ was used to construct a PPI network for the intersecting genes. Secondly, genes with relatively high connectivity were extracted from the overall network using the CytoHubba plugin in Cytoscape.

### 2.6 Gene ontology (GO) and Kyoto encyclopedia of genes and genomes (KEGG) functional enrichment analysis

To explore the potential roles of AD-related genes, GO and KEGG functional enrichment analyses were performed on the candidate genes. GO analysis encompasses three categories: biological process (BP), cellular component (CC), and molecular function (MF), while KEGG analysis identifies pathways of interactions between genes. The ‘clusterProfiler’ and ‘Org.Hs.eg.db’ packages in the R platform were used to perform these analyses, allowing for a deeper investigation of the underlying mechanisms involved in the occurrence and development of AD.

### 2.7 Machine learning

Through the PPI network, 21 candidate genes associated with AD were identified. To further refine the list of potential genes for AD, three machine learning techniques were applied to these 21 candidate genes (Akkaya and Kalkan, 2023). The ‘glmnet’, ‘e1071’, ‘Boruta’, and ‘xgboost’ packages in the R platform were used to implement the Least Absolute Shrinkage and Selection Operator (LASSO), Support Vector Machine-Recursive Feature Elimination (SVM-RFE), Boruta, and Extreme Gradient Boosting (XGBoost) algorithms. The common intersections from these methods were then used to identify the final candidate genes for AD (Friedman et al., 2010; Zhang et al., 2024; Elith et al., 2008; McCutcheon et al., 2025).

### 2.8 Construction and validation of logistic regression

Regression analysis was conducted on the candidate genes in the training set GSE63060 and the validation set GSE63061 using the SPSS 27.0 statistical program to construct a robust combinatory model. A *p*-value of <0.05 was considered statistically significant. The receiver operating characteristic (ROC) curve was used to assess and measure the area under the curve (AUC) to evaluate the diagnostic potential of the candidate genes and identify the hub gene (Zhao et al., 2023). Data analysis and visualization were performed using the ‘pROC’ package in R software (Robin et al., 2011).

### 2.9 Construction and validation of nomograms

A nomogram, also referred to as a calibration chart, is based on multivariate regression analysis and integrates multiple predictive indicators. It employs scaled segments plotted on a common plane according to a specific ratio, representing the relationships between various variables in a predictive model (Fu et al., 2024). In R software, the ‘rms’ package is used to integrate the Hub gene feature data and construct the nomogram, while the ‘rmda’ package is employed to plot the clinical decision curve (DCA) (Vickers and Elkin, 2006).

### 2.10 Immune cell infiltration analysis

Immune infiltration analysis aims to assess the proportion of different immune cells in the human microenvironment and explore which immune cell types play a crucial role in the onset and progression of the disease. CIBERSORT is a deconvolution algorithm based on linear support vector regression, which estimates the proportion of immune cells by deconvolving the expression matrix of immune cell subtypes. In this study, the CIBERSORT deconvolution algorithm was employed to obtain the scores for immune cell infiltration. Box plots were used to visualize the differences in immune cell infiltration between AD patients and healthy controls. The ‘CIBERSORT’ package in R software facilitated both qualitative and quantitative analysis of the matrix. Additionally, the ‘tidyverse’ package was used to visualize the correlation between Hub genes and immune cell subpopulation infiltration.

### 2.11 Regulatory network analysis

The candidate genes were entered into three miRNA prediction databases (miRTarBase, TarBase, and miRecords) on the NetworkAnalyst website^3^ to identify gene-related miRNAs. Additionally, the four transcription factor (TF) prediction databases (TRRUST, Summary, JASPAR, and ChEA3) on the NetworkAnalyst website were used to obtain gene-related TFs. Finally, Cytoscape software was employed to construct the Gene-TF and Gene-miRNA regulatory networks.

### 2.12 Concentration expression and diagnostic efficacy of serum c-Myc in AD patients

#### 2.12.1 Research object

The subjects of this study were patients who visited the Second Affiliated Hospital of Harbin Medical University between October 2023 and November 2024. A total of 82 subjects were included, with 41 individuals in the AD group and 41 in the control group. AD patients met the 2011 NIA-AA core diagnostic criteria for suspected AD dementia. The study was approved by the Ethics Committee of the Second Affiliated Hospital of Harbin Medical University, and informed consent was obtained from all participants. General information, including name, gender, age, and medical record number, was recorded, along with laboratory blood markers such as apolipoprotein B, lipoprotein(a), total cholesterol, triglycerides, high-density lipoprotein, low-density lipoprotein, isotype Cystine, and apolipoprotein AI. Cognitive function was assessed using the Mini-Mental State Examination (MMSE).

#### 2.12.2 Blood sample collection and measurement

Collect 10 mL of fasting venous blood from the experimental subjects, then centrifuge at 1000 × g for 20 min at a temperature of 2–8°C. After centrifugation, transfer the serum to an enzyme-free, sterile EP tube and store it at −80°C for future analysis. Prior to the experiment, thaw the serum at room temperature and allow the plasma to recover. Serum c-Myc concentration will be measured according to the instructions provided with the human c-Myc enzyme-linked immunosorbent assay (ELISA) kit (ZC-31835) from Shanghai Zhuocai Biotechnology Co., Ltd., using a microplate reader at a wavelength of 450 nm.

### 2.13 Statistical analysis

The data were analyzed using SPSS 27.0 statistical software. General information and laboratory blood indicators are presented as mean ± standard deviation if they follow a normal distribution, or as median (interquartile range) if they follow a skewed distribution. Pearson correlation analysis was performed on serum c-Myc levels, and the diagnostic value of serum c-Myc for AD was assessed using the ROC curve. Statistical significance was set at *p* < 0.05 (*p < 0.05; **p < 0.01; ***p < 0.001).

## 3 Results

### 3.1 Analysis of Alzheimer’s disease biomarkers based on bioinformatics

#### 3.1.1 Identification of differentially expressed genes

**Figure 1:**
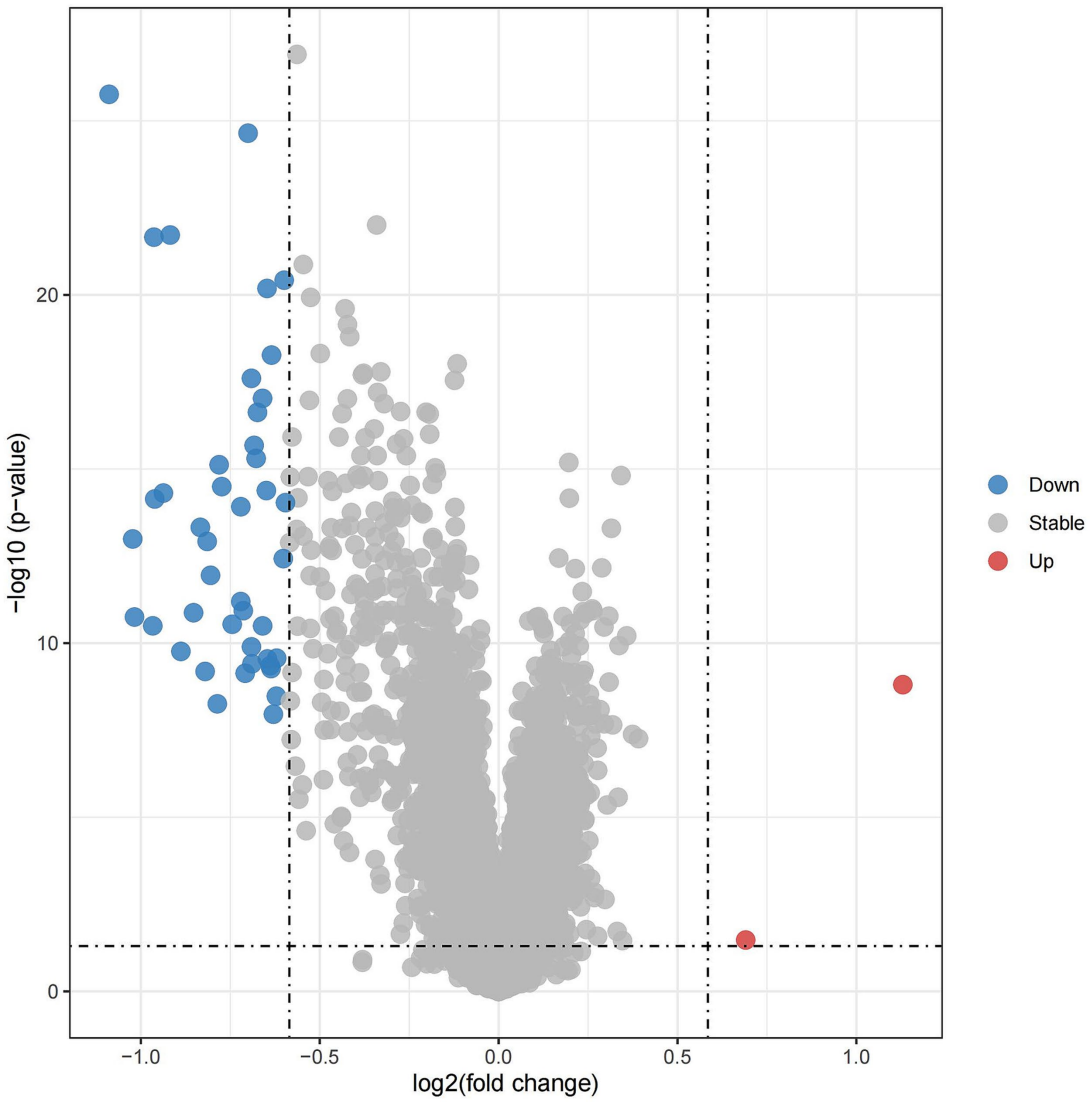
The volcano plot of GSE63060 differentially expressed genes.

The screening criteria for the GSE63060 dataset included differential gene expression analysis with a log2|fold change| > 0.585 and *p* value < 0.05. Genes with a log2 fold change > 0.585 and *p* value < 0.05 were classified as up-regulated, while genes with a log2 fold change < −0.585 and *p* value < 0.05 were classified as down-regulated. A total of 45 DEGs were identified between AD and healthy controls. Of these, 43 genes were down-regulated (represented in blue), 2 genes were up-regulated (represented in red), and the remaining genes showed no significant difference (represented in gray) (Figure 1).

#### 3.1.2 Constructing a WGCNA to identify key modules associated with AD

**Figure 2:**
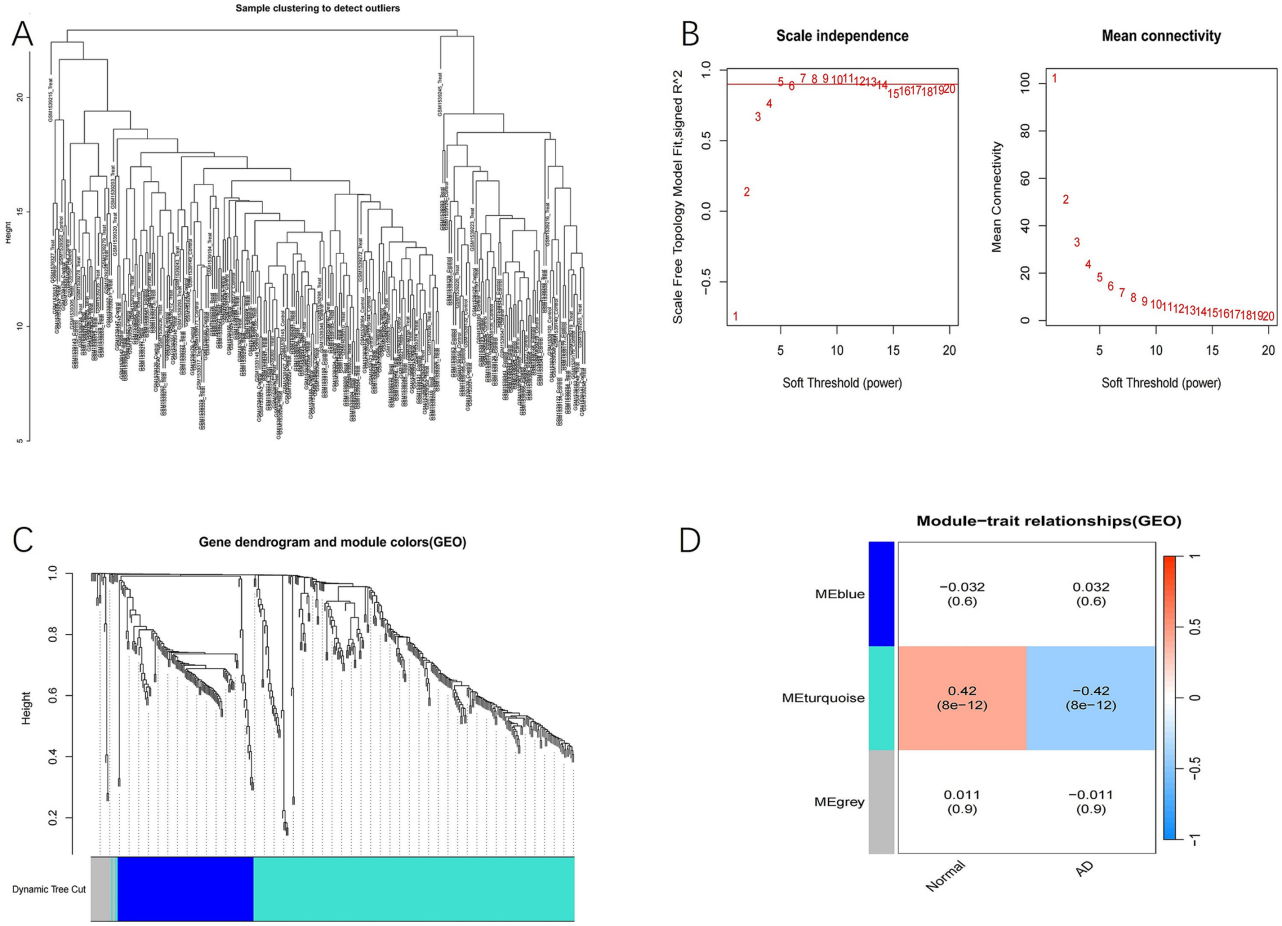
Identification of important AD modules in WGCNA. **(A)** Module clustering difference diagram. The shorter the height, the more similar the two modules are. **(B)** Selection of soft threshold. **(C)** Tree module diagram. **(D)** Correlation matrix diagram of each module. Pink indicates positive correlation and blue indicates negative correlation.

Firstly, a module clustering difference graph (Figure 2A) was generated to filter out genes with minimal expression changes in the GSE63060 dataset and to identify any outliers. To ensure a scale-free distribution, the adjacency matrix weight parameter (power) was set to 5 (Figure 2B). A clustering dendrogram was then constructed (Figure 2C), grouping the genes into three modules: blue (*p* = 0.6), turquoise (*p* = 8e−12), and gray (*p* = 0.9) (Figure 2D). Based on Pearson correlation, the turquoise module, which exhibited the strongest association with AD, was selected for further bioinformatics analysis.

#### 3.1.3 Intersection genes

**Figure 3:**
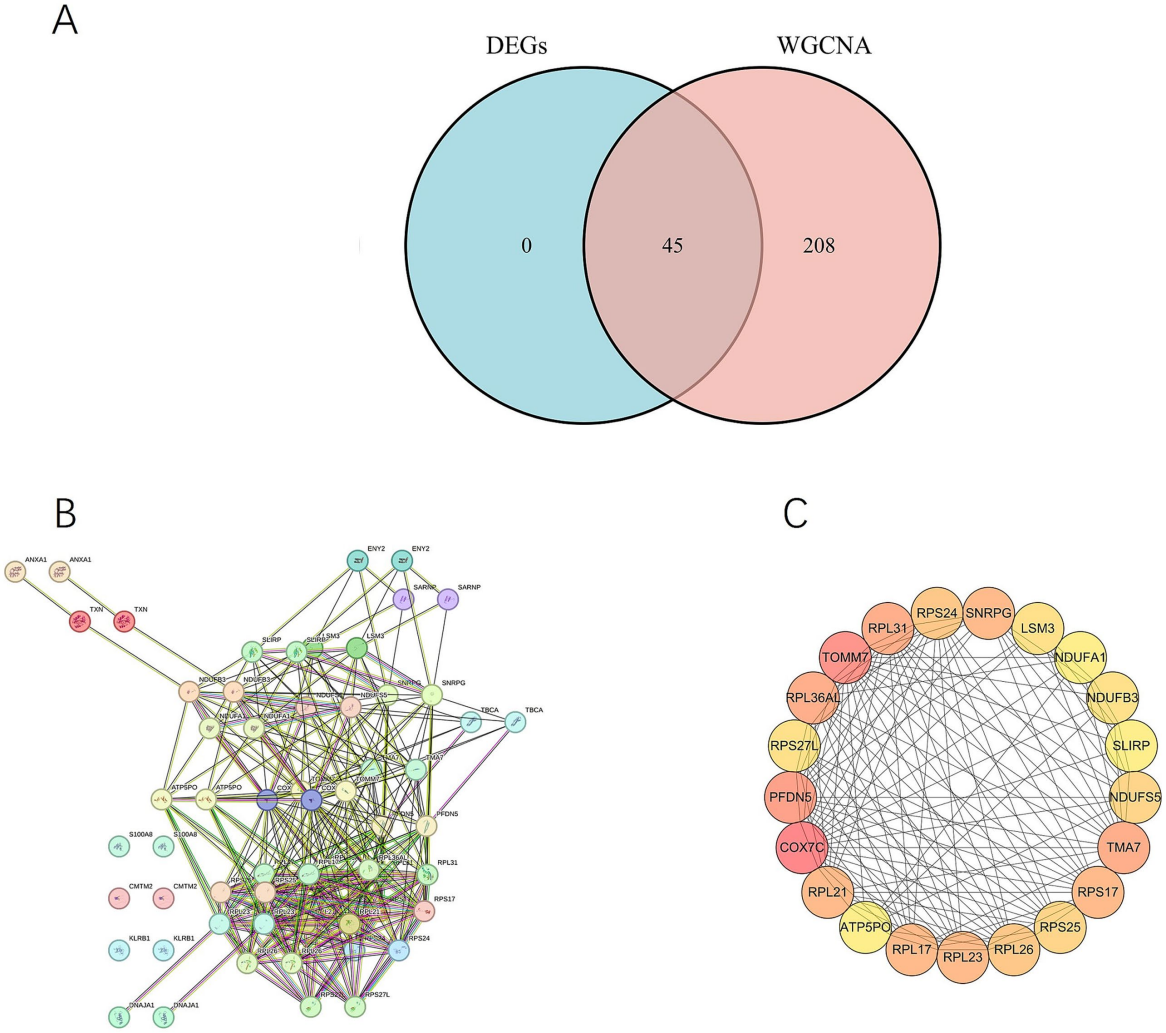
**(A)** Intersection of differentially expressed genes (DEGs) and the WGCNA turquoise module. **(B)** PPI network diagram. **(C)** The size of the degree reflects the centrality of the node in the network. The darker the color, the higher the degree score.

The ‘venn.diagram’ package was used to intersect the DEGs with the 253 genes in the WGCNA turquoise module, resulting in 45 intersection genes (Figure 3A).

#### 3.1.4 Protein network interactions

PPI network analysis was conducted on the 45 intersection genes using the STRING database. The core network consisted of 30 nodes and 147 edges, where each node represents a differentially expressed gene and each edge denotes a gene interaction (Figure 3B). The resulting data in TSV format were imported into Cytoscape for network visualization, and the degree algorithm in CytoHubba was employed to assess the significance of each node. After reviewing the literature, the top 21 genes were selected based on their degree ranking for further analysis: COX7C, TOMM7, PF DN5, TMA7, RPL31, RPL36AL, RPS17, SNRPG, RPL23, RPL17, RPL21, RPS24, RPL26, RPS25, NDUFS5, RPS27L, LSM3, NDUFB3, SLIRP, NDUFA1, and ATP5O (Figure 3C).

#### 3.1.5 Functional and pathway enrichment analysis

**Figure 4:**
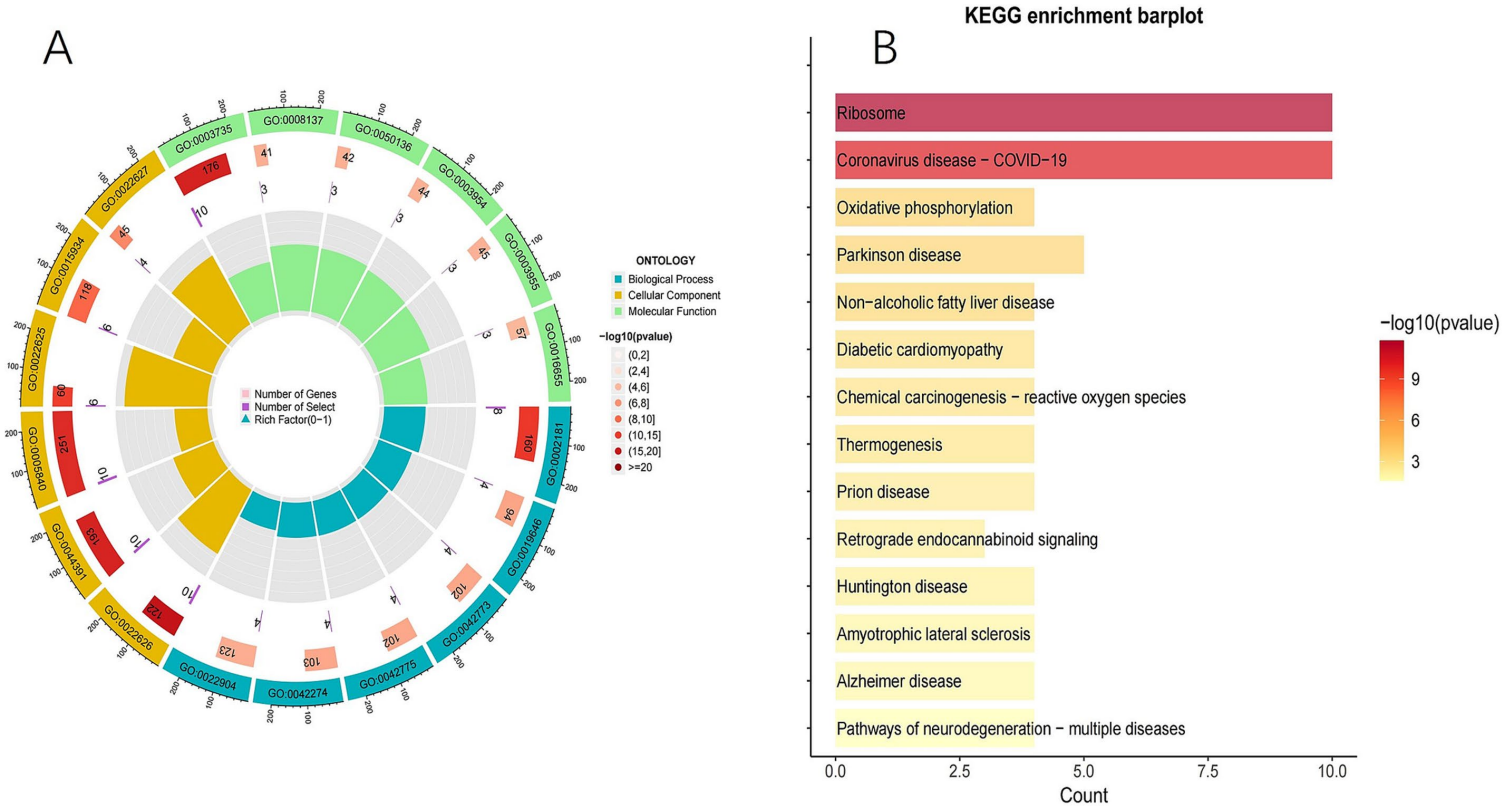
**(A)** GO enrichment analysis. From the outside to the inside, the first circle is the GO id (pathway id) label; the bar length of the second circle corresponds to the number of background genes, the depth of the color corresponds to the significance level, and *p*-value; the third circle corresponds to the number of target genes; the fourth circle (Polar histogram) is the enrichment factor. **(B)** KEGG enrichment analysis.

To determine the functions of the genes in the GSE63060 dataset, this study performed GO and KEGG enrichment analyses on the 21 identified genes. In GO analysis, the BP were significantly enriched in cytoplasmic translation, electron transport, and ribosome biogenesis; the CC were enriched in structures such as ribosomes and respiratory chain complexes; and the MF were enriched in ribosomal structural components, NADH dehydrogenase activity, and oxidoreductase activity (Figure 4A). In KEGG analysis, the pathways were predominantly enriched in the ribosome pathway, novel coronavirus pathway, AD, and other key signaling pathways (Figure 4B).

#### 3.1.6 Screening for novel biomarkers for the diagnosis of AD using machine learning

**Figure 5:**
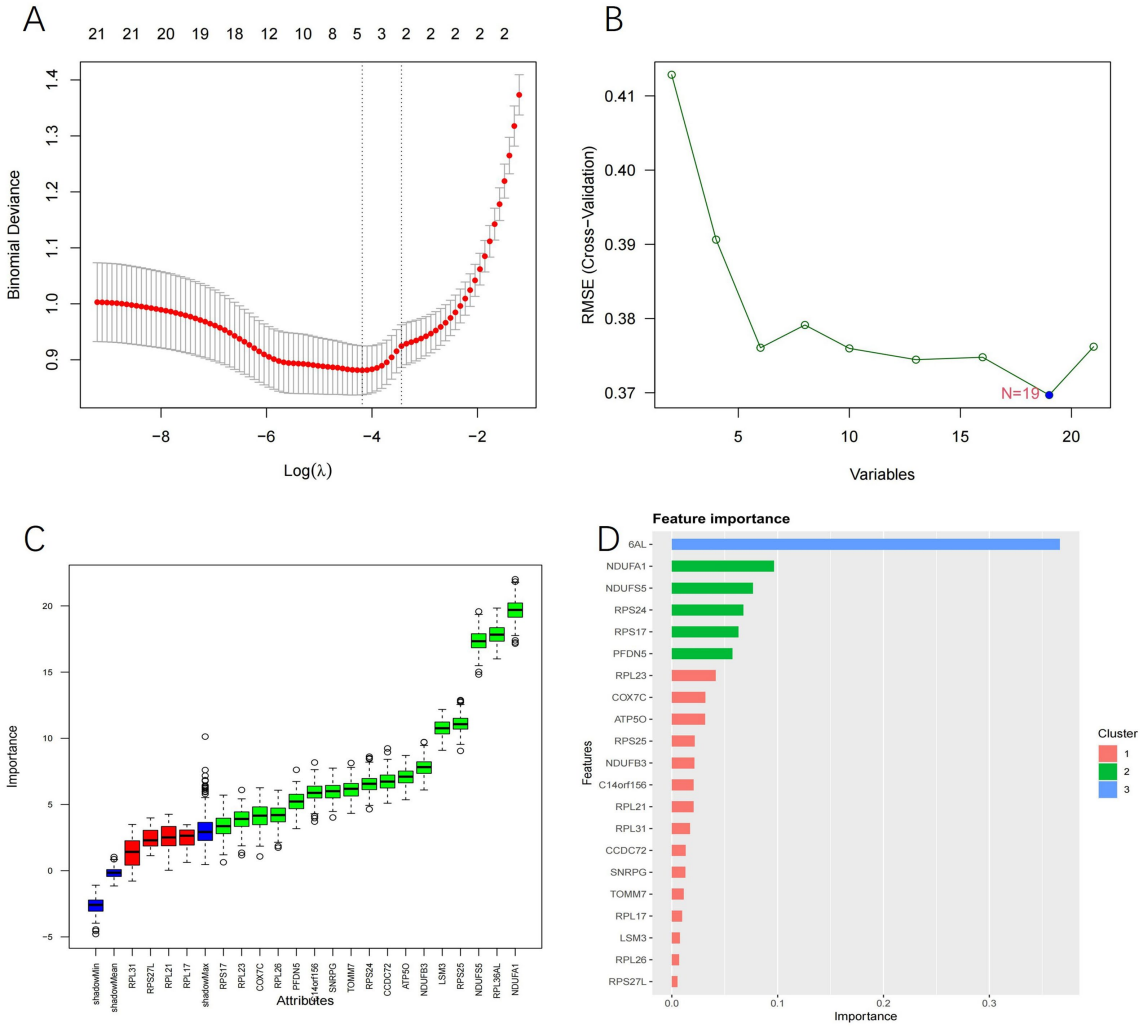
**(A)** LASSO regression analysis results. **(B)** SVM-RFE analysis results. **(C)** Boruta analysis results. **(D)** XGboost analysis results.

To further identify genes associated with the disease, feature selection was performed on the 21 genes using LASSO regression, the SVM-RFE algorithm, Boruta algorithm, and XGBoost algorithm. LASSO regression analysis identified 5 characteristic genes (Figure 5A), the SVM algorithm identified 19 characteristic genes (Figure 5B), the Boruta algorithm identified 17 characteristic genes (Figure 5C), and the XGBoost algorithm identified 21 characteristic genes (Figure 5D). The characteristic genes identified by the four machine learning algorithms were intersected, resulting in the identification of 5 candidate genes for subsequent research: RPL36AL, NDUFA1, NDUFS5, RPS25, and COX7C.

#### 3.1.7 Establishment and verification of classification diagnostic models

**Figure 6:**
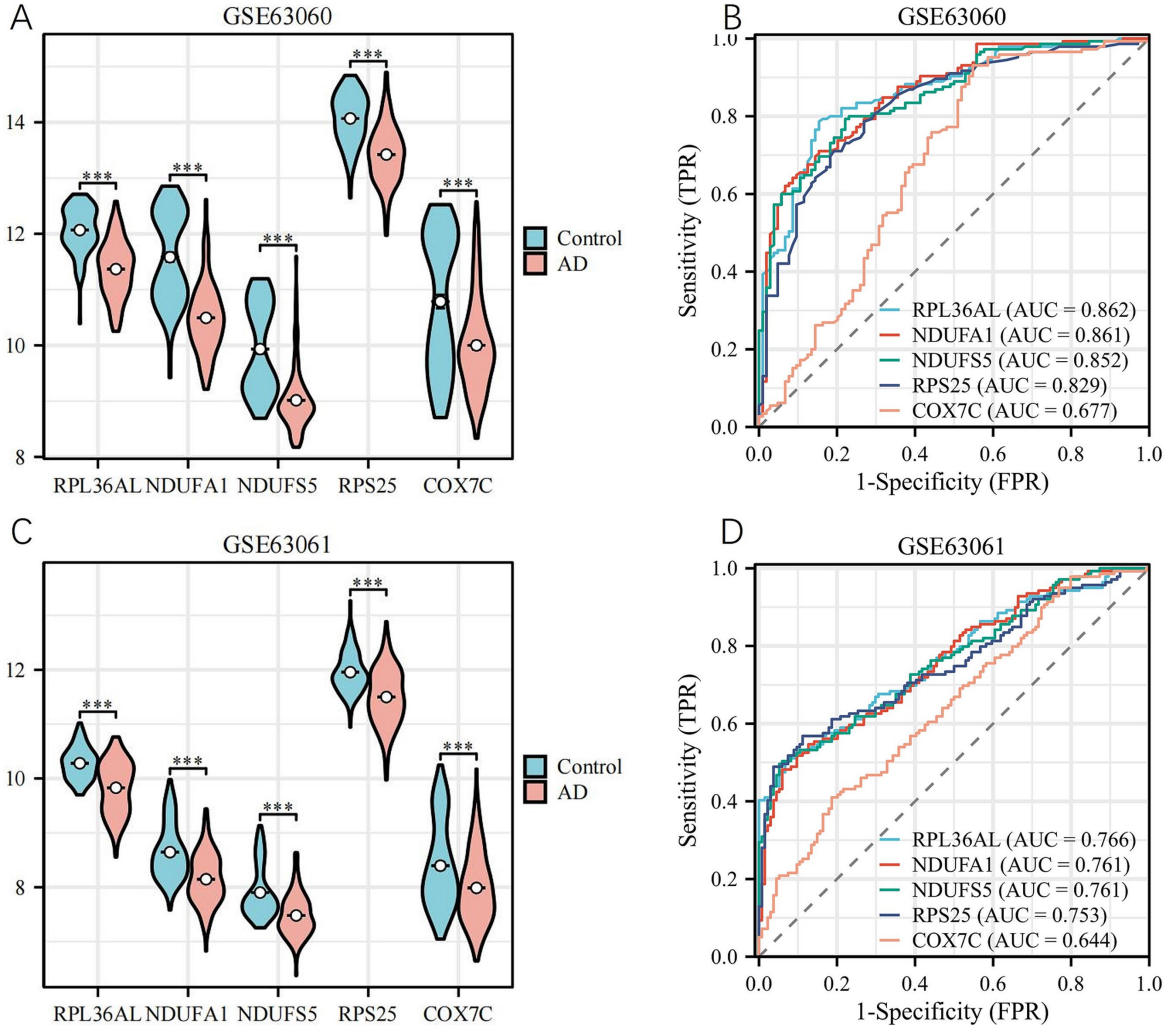
Expression levels and ROC curves of GSE63060 and GSE63061 **(A)** Expression levels of key genes in GSE63060. **(B)** ROC curve of key genes in GSE63060. **(C)** Expression levels of key genes in GSE63061. **(D)** ROC curve of key genes in GSE63061. *, *p* < 0.05; **, *p* < 0.01; ***, *p* < 0.001.

To determine the expression levels of the candidate genes in AD patients, regression analysis was performed on the GSE63060 and GSE63061 datasets. The results of the expression analysis indicated that all five genes were significantly associated with AD (*p* < 0.05) (Figures 6A,C). Specifically, RPL36AL, NDUFA1, NDUFS5, RPS25, and COX7C were found to be downregulated in the blood of AD patients and upregulated in the blood of healthy individuals. The expression patterns of these five genes were consistent across both the training set and the validation set.

ROC curves were plotted to evaluate the predictive performance and diagnostic value of the genes for Alzheimer’s disease. The results are shown in Figures 6B,D. In the training set (GSE63060), the AUC values were as follows: RPL36AL (AUC = 0.862, *p* < 0.05), NDUFA1 (AUC = 0.861, *p* < 0.05), NDUFS5 (AUC = 0.852, *p* < 0.05), RPS25 (AUC = 0.829, *p* < 0.05), and COX7C (AUC = 0.677, *p* < 0.05). In the validation set (GSE63061), the AUC values were: RPL36AL (AUC = 0.766, *p* < 0.05), NDUFA1 (AUC = 0.761, *p* < 0.05), NDUFS5 (AUC = 0.761, *p* < 0.05), RPS25 (AUC = 0.753, *p* < 0.05), and COX7C (AUC = 0.644, *p* < 0.05). In terms of biological mechanisms, previous studies have shown that these genes are associated with the structure and function of AD ribosomes (Suzuki et al., 2022; Zhuang et al., 2023). Furthermore, through bioinformatics analysis, we have discovered that these genes exhibit high connectivity within biochemical networks. Ultimately, RPL36AL, NDUFA1, NDUFS5, and RPS25 were identified as AD-related hub genes.

#### 3.1.8 Nomotu

**Figure 7:**
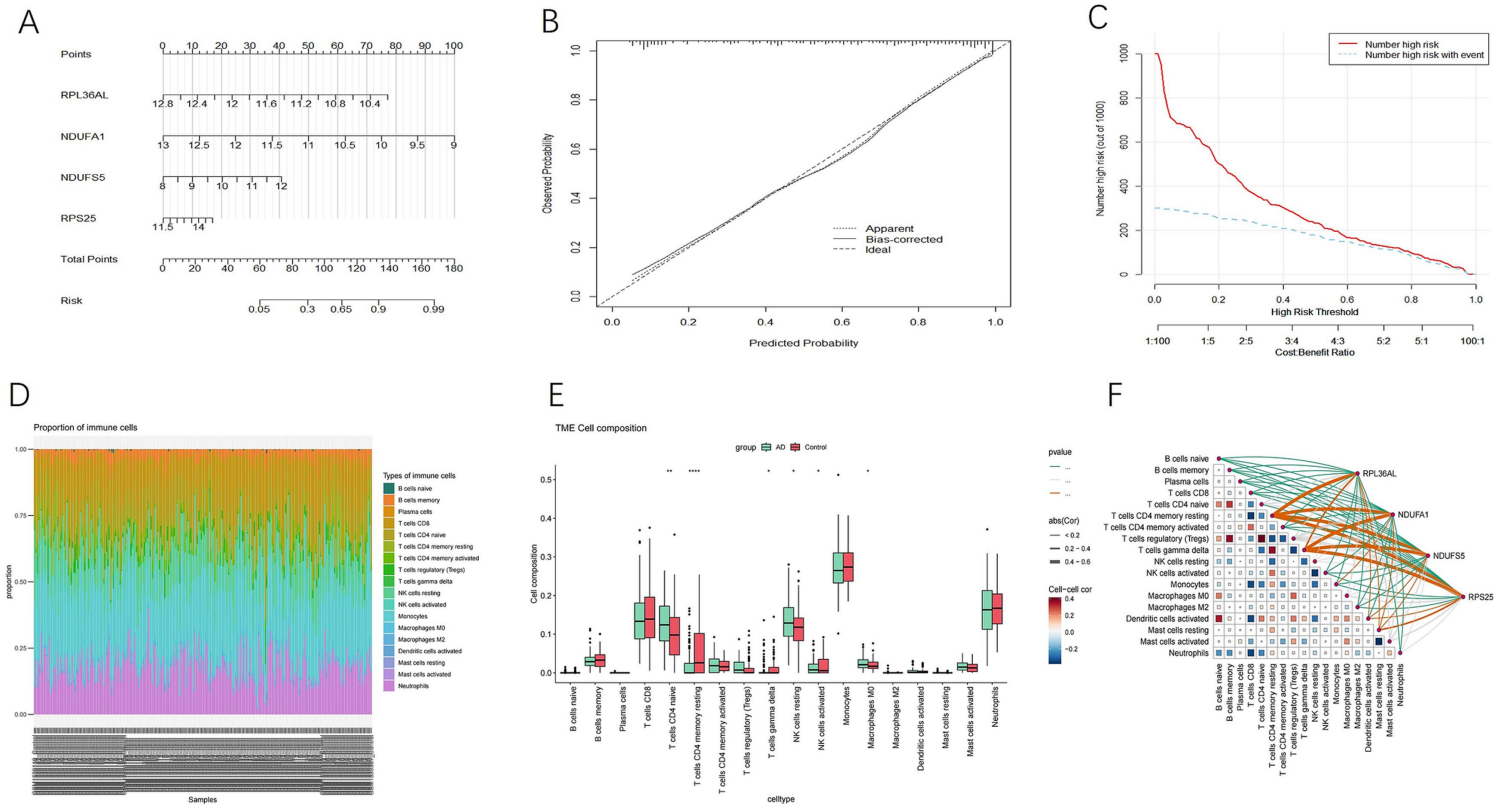
**(A)** Nomogram. **(B)** Calibration curve. **(C)** Decision analysis curve. **(D)** Proportion of immune cells in 22. **(E)** Box plot of immune cells between AD group and control group. **(F)** Heat map of the correlation between Hub genes and immune cells.

In the nomogram, each variable axis represents the value of a gene, and the number of points corresponding to each gene value is determined based on the upward straight line. The total points axis represents the sum of the relevant points (Figure 7A). The calibration curve indicates that the predicted values align closely with the observed values, confirming the accuracy of the gene prediction results (Figure 7B). The decision analysis curve shows a higher net benefit over the ‘treat all’ and ‘no treat’ strategies within a specific high-risk threshold range (approximately 0.2 to 0.6) (Figure 7C). Therefore, the model demonstrates practical value for clinical decision-making within this threshold range.

#### 3.1.9 Immune cell analysis

The immune cell abundance of 22 immune cell types in AD patients and healthy controls was analyzed using Cibersort (Figure 7D). The results are presented as boxplots (Figure 7E). In AD patients, the proportions of initial B cells, resting memory CD4 + T cells, activated natural killer (NK) cells, and monocytes were lower compared to those in healthy individuals. Conversely, the proportions of plasma cells, initial CD4 + T cells, regulatory T cells, gamma delta T cells, resting NK cells, macrophages M2, and activated mast cells were higher in AD patients. Specifically, initial CD4 + T cells and resting memory CD4 + T cells showed a significant difference (*p* < 0.01), while resting NK cells, activated NK cells, macrophages M0, and gamma delta T cells also showed a significant difference (*p* < 0.05). These findings suggest that naïve CD4 + T cells and resting memory CD4 + T cells may play key roles in the immune response in AD.

In the analysis of the correlation between immune cell subpopulations and gene expression, we constructed a network diagram to illustrate the complex relationships (Figure 7F). By calculating the correlation coefficients, we found that RPL36AL, NDUFA1, NDUFS5, and RPS25 were positively correlated with resting memory CD4 + T cells and gamma delta T cells, while they were negatively correlated with activated NK cells.

#### 3.1.10 Regulatory network

**Figure 8:**
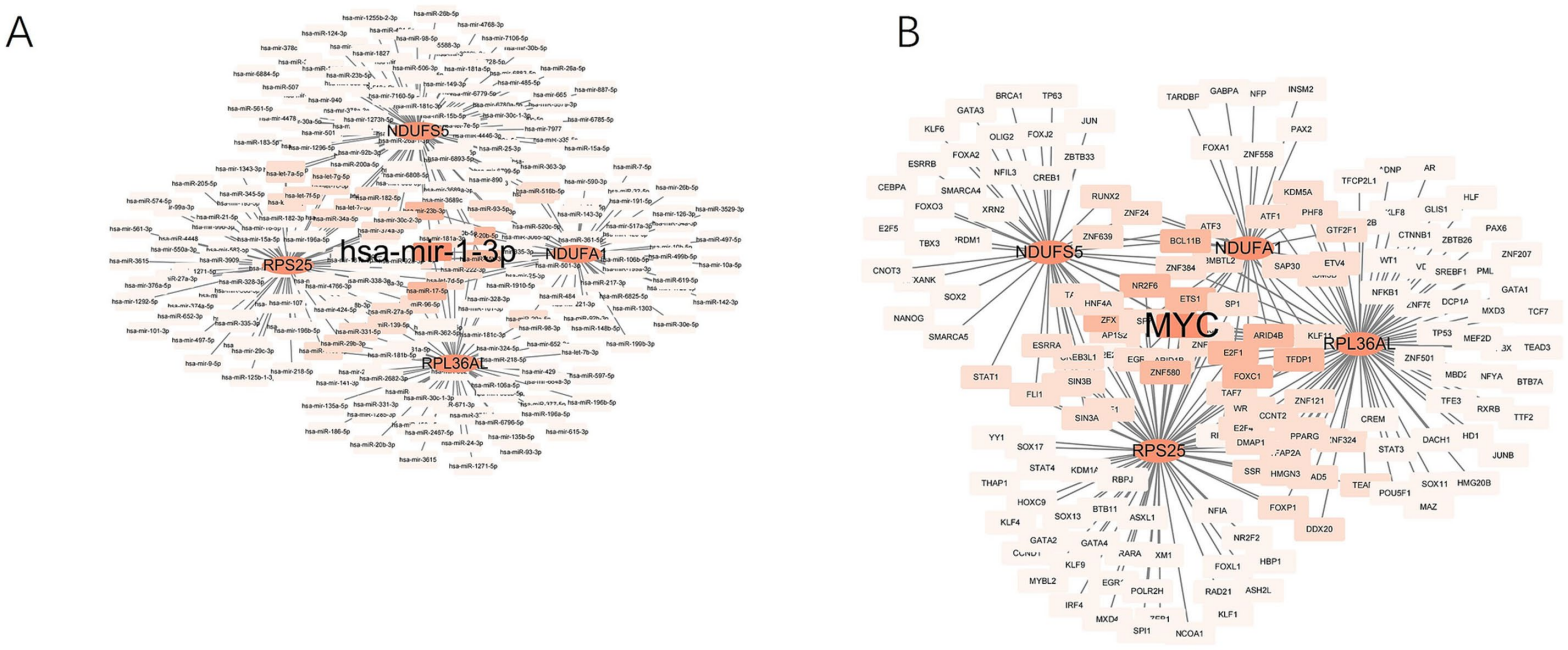
TF-gene, Gene-miRNA regulatory network **(A)** Gene-miRNA. **(B)** TF-gene.

In the miRNA prediction analysis, we searched for miRNAs associated with RPL36AL, NDUFA1, NDUFS5, and RPS25 and constructed a Cytoscape network. It was found that all four genes share a common miRNA, has-miR-1-3p (Figure 8A). For TF prediction, the upstream TFs of RPL36AL, NDUFA1, NDUFS5, and RPS25 were identified. After constructing the Cytoscape network, we observed that all four genes share a common TF, namely Myc, also known as c-Myc (Figure 8B).

### 3.2 Differences in serum c-Myc concentration between AD patients and controls

Through bioinformatics analysis, we identified c-Myc as a common transcription factor for RPL36AL, NDUFA1, NDUFS5, and RPS25. To identify potential biomarkers for AD through a simple and practical method like blood ELISA, we decided to focus on c-Myc in clinical experiments. C-Myc, an important oncogene, has been found to be significantly elevated in conditions such as gastric cancer and rheumatoid arthritis. However, the specific expression level of c-Myc protein in the serum of AD patients remains underexplored. This study aims to evaluate the concentration differences of c-Myc in the serum of AD patients versus healthy individuals and assess its diagnostic potential, offering a new serodiagnostic marker for AD in clinical settings.

#### 3.2.1 Comparison of general information and laboratory blood indicators between the AD group and the control group

After collecting the general information and laboratory blood indicators for both the AD group and the control group, we performed regression analysis (Table 1). The chi-square test was used to assess gender differences. The statistical analysis revealed no significant difference in gender or age between the two groups (*p* > 0.05). In terms of laboratory blood test indicators, apolipoprotein AI, apolipoprotein B, lipoprotein a, total cholesterol, triglycerides, and low-density lipoprotein did not show significant differences (*p* > 0.05). However, high-density lipoprotein and isotype cysteine were found to have statistically significant differences (*p* < 0.05).

**Table 1:** Comparison of general information and laboratory blood indicators between the AD group and the control group.

Variables	Control group (*N* = 41)	AD group (*N* = 41)	*X^2^*/Z/t	*p-*value
MMSE^1^	30	19.15 ± 4.45	/	/
Educational	12 (9.00, 13.00)	12 (9.00, 15.00)	−1.234	0.217
Age (year)	67.0 ± 7.1	67.6 ± 9.0	0.326	0.745
man/female (*N*)	14/27	10/31	0.943	0.332
Apolipoprotein AI (g/L)	1.39 ± 0.31	1.752 ± 1.186	1.899	0.630
Apolipoprotein B (g/L)	0.96 (0.82, 1.06)	0.94 (0.75, 1.11)	−0.366	0.714
Apolipoprotein a (nmom/L)	28.89 ± 40.67	46.93 ± 76.58	1.332	0.187
Total cholesterol (mmol/L)	4.65 (4.07, 5.29)	4.74 (4.00, 5.46)	−0.236	0.813
Triglycerides (mmol/L)	1.69 ± 0.97	1.51 ± 0.69	−0.948	0.346
HDL (mmol/L)	1.24 (1.00, 1.61)	1.38 (1.18, 1.81)	−2.041	0.041*
LDL (mmol/L)	2.73 (2.34, 3.41)	2.77 (2.24, 3.32)	−0.218	0.827
Homocysteine (umol/L)	10.31 (8.58, 12.17)	12.08 (9.50, 14.55)	−2.292	0.022*

HDL, High-Density Lipoprotein; LDL, Low-Density Lipoprotein.

^1^The MMSE in the control group is 0, and statistical analysis cannot be performed, *p < 0.05.

#### 3.2.2 Comparison of serum c-Myc concentration between AD group and control group

**Figure 9:**
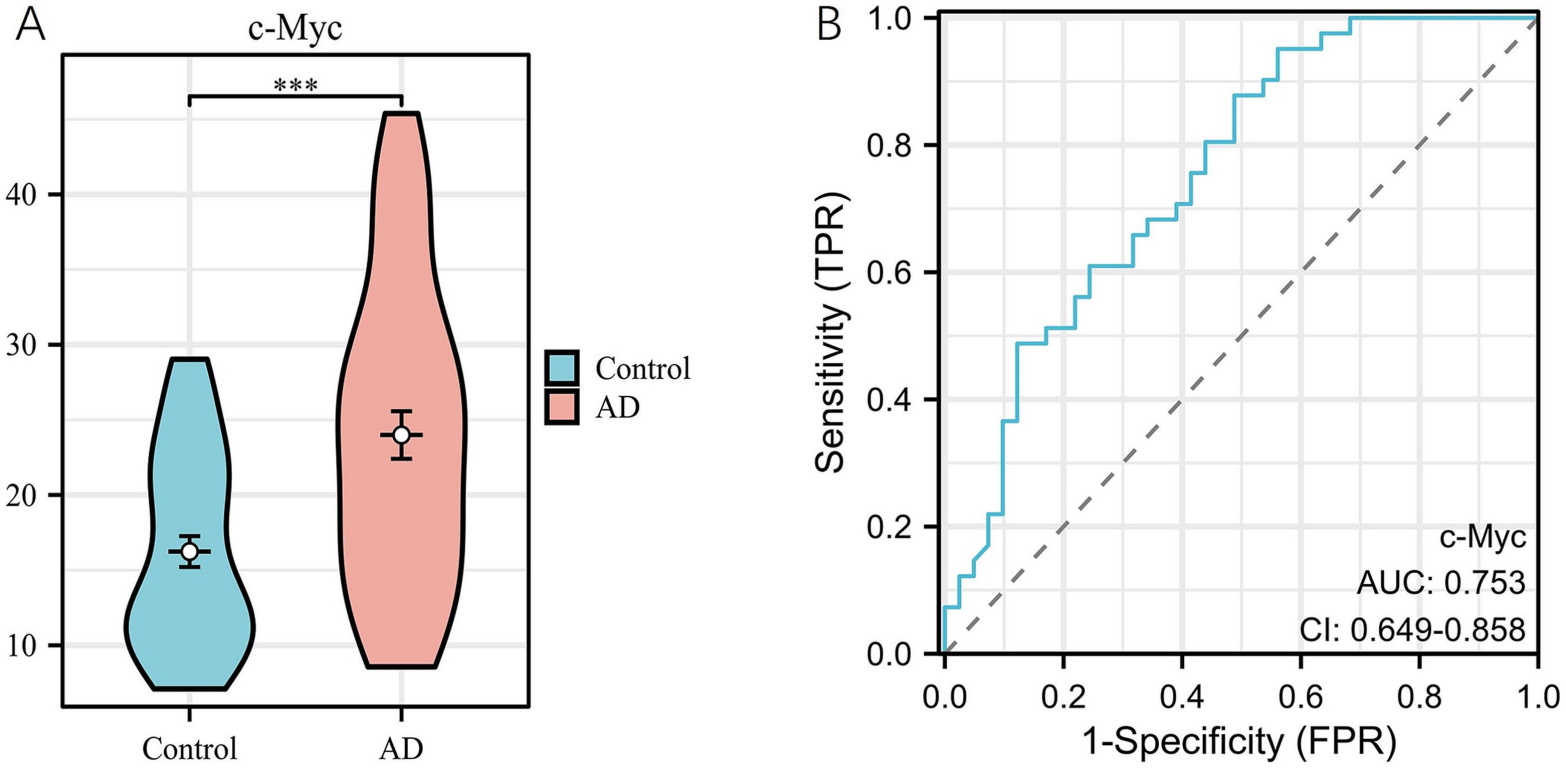
**(A)** Comparison of serum c-Myc concentration between AD group and control group. **(B)** ROC curve of serum c-Myc.

Comparison between the two groups revealed that the serum c-Myc concentration in the AD group was significantly higher than in the control group, with statistical significance (*p* < 0.05) (Figure 9A). The median serum c-Myc concentration in the AD group was 23.4 ng/mL, while the median concentration in the control group was 14.1 ng/mL (Table 2).

**Table 2:** Comparison of serum c-Myc concentration between AD group and control group.

Variable	Control group (*N* = 41)	AD group (*N* = 41)	*X^2^*/*Z*/*t*	*p-*value
c-Myc (ng/ml)	14.1 (10.9, 21.3)	23.4 (15.8, 30.2)	3.571	<0.001^✳✳✳^

***p < 0.001.

#### 3.2.3 ROC curve analysis of serum c-Myc between AD group and control group

The serum c-Myc concentration had an AUC of 0.753 (95% CI: 0.649–0.858), with a cutoff value of 22.955. The sensitivity for predicting the occurrence of AD was 0.878, while the specificity was 0.512 (Figure 9B; Table 3). These results indicate that there is a correlation between elevated serum c-Myc levels and AD.

**Table 3:** ROC curve of serum c-Myc between AD group and control group.

Index	AUC (95% CI)	Cutoff	Sensitivity	Specificity	Youden index
c-Myc	0.753 (0.649–0.858)	22.955	0.878	0.512	0.390

## 4 Discussion

As the global population ages, AD has become an increasingly critical issue. It not only places a significant economic burden on families but also contributes to a substantial socioeconomic strain on entire nations. Currently, the diagnosis of AD is based on multiple factors, and there is no single, definitive method for confirmation. Given that AD is an irreversible disease, it is crucial to develop better approaches to address the challenges it presents and to improve the quality of life for affected patients.

In this study, five candidate genes—RPL36AL, NDUFA1, NDUFS5, RPS25, and COX7C—were identified through PPI and machine learning techniques. After constructing a classification diagnostic model, it was found that COX7C exhibited moderate diagnostic performance. As a result, RPL36AL, NDUFA1, NDUFS5, and RPS25 were selected as hub genes for further analysis.

Previous studies have consistently demonstrated that RPL36AL (Ji et al., 2022), NDUFA1 (Li et al., 2018), NDUFS5 (Zhuang et al., 2023; Yan et al., 2024), RPS25 (Suzuki et al., 2022), and COX7C (Wang et al., 2021) are associated with mitochondrial function and ribosomal structure in AD. RPL36AL encodes a component of the ribosomal 60S subunit and is located on chromosome 6q22.1 in humans (Hountondji et al., 2012). In immune infiltration analysis of AD, RPL36AL has been identified as a potential diagnostic marker for AD (Li et al., 2022). NDUFA1 and NDUFS5 are both protein-coding genes located on the human X chromosome at q24 and on chromosome 1 at 1p34.3, respectively. These genes primarily encode accessory subunits of respiratory chain complex I, playing crucial roles in the maintenance of mitochondrial function and cellular energy production (Fernandez-Moreira et al., 2007; Loeffen et al., 1999). The Gly32Arg SNP mutation in NDUFA1 may be involved in the development of dementia praecox (Huttula et al., 2022). The relationship between NDUFA1, NDUFS5, and AD remains unclear. However, it is well established that mutations or functional abnormalities in NDUFA1 and NDUFS5 can lead to mitochondrial dysfunction, which has been confirmed through various studies as a key pathogenic mechanism in AD. This suggests that NDUFA1 and NDUFS5 may contribute to AD pathogenesis through their impact on mitochondrial function. RPS25 encodes a highly basic protein that is part of the ribosomal 40S subunit. It is located on the q23.3 region of human chromosome 11 and belongs to the S25E family of ribosomal proteins (Kubota et al., 1999). Masayoshi Suzuki et al. demonstrated through proteomic analysis of brain capillaries in AD that the expression of RPS25 was up-regulated, which indirectly suggests that RPS25 is involved in the onset and progression of AD (Suzuki et al., 2022). COX7C is located on human chromosome 5 and encodes a component of mitochondrial respiratory chain complex IV, playing a role in cellular energy metabolism (Wu et al., 2020). In a study involving 17 COX-related genes and 1,572 individuals of Han Chinese descent, certain mutations in COX7C were found to be associated with AD, suggesting that COX7C may contribute to the pathogenesis of AD (Bi et al., 2018).

Immune cell infiltration plays a crucial role in the development of AD. This study found that initial CD4 + T cells and resting memory CD4 + T cells were most significantly expressed in AD. In the analysis of the correlation between immune cell subpopulations and gene expression, resting memory CD4 + T cells and gamma delta T cells showed a significant positive correlation with RPL36AL, NDUFA1, NDUFS5, and RPS25. As key components of the adaptive immune system, T cells may be involved in the pathogenesis of AD. Previous studies have shown that T cell subpopulations change in the cerebrospinal fluid and blood of AD patients, and peripheral CD4 + T cells can cross the blood–brain barrier, contributing to the pathological formation of AD (Guo et al., 2023). The role of CD4 + T cells in AD remains unclear, with uncertainties about whether they play a protective or detrimental role. However, it is well-established that T cells are involved in the onset and progression of AD (McManus et al., 2015).

After comparing the general data, we observed that high-density lipoprotein (HDL) levels were elevated in AD patients compared to the control group. HDL in AD patients exhibits anti-inflammatory and antioxidant properties and plays a role in clearing Aβ, which may potentially delay neurodegeneration (Button et al., 2019). However, some studies suggest that HDL levels may be negatively correlated with cognitive function, with excessive elevation potentially exacerbating the pathological process. This indicates that the detrimental effects of HDL could outweigh its protective benefits. Moreover, comorbidities commonly seen in AD patients, such as diabetes and cardiovascular disease, may indirectly influence HDL levels. Additionally, lifestyle factors like low education levels and a lack of exercise may affect HDL through metabolic pathways. Therefore, the role of HDL in AD requires further investigation, particularly regarding its specific molecular mechanisms and how it interacts with different stages of the disease.

The results indicated that the blood c-Myc concentration was significantly elevated in AD patients. The median serum c-Myc concentration in the AD group was 23.4 ng/mL, while in the control group, it was 14.1 ng/mL, showing a statistically significant difference. c-Myc is a key transcription factor involved in cell cycle activation and plays a crucial role in regulating cell proliferation and apoptosis (Li et al., 2020). This study innovatively conducted an ELISA experiment to measure c-Myc levels in the serum of AD patients and healthy controls for the first time. The results demonstrated that c-Myc holds significant diagnostic and predictive value, suggesting its potential involvement in the onset and progression of AD. Additionally, this study provides a new starting point for AD research and indirectly supports the notion that the four hub genes could serve as potential biomarkers for AD.

This study has certain limitations, primarily centered around the reliance on bioinformatics, with further experimental validation still required. Additionally, the AD inclusion criteria did not include A*β* measurement, which may have introduced some biases. Furthermore, only 41 AD cases were included in the serological study of c-Myc, limiting the robustness of the findings. To enhance the diagnostic specificity of this biomarker for AD, future work will involve multi-omic cross-validation, optimization of algorithms and models, expansion of the sample size, and conducting relevant *in vivo* and *in vitro* experiments.

## 5 Conclusion

This study suggests that RPL36AL, NDUFA1, NDUFS5, and RPS25 may serve as potential biomarkers for the diagnosis of AD. Furthermore, serum c-Myc shows considerable promise as a biomarker for the early diagnosis of AD.

## Data Availability

The original contributions presented in the study are included in the article/supplementary material, further inquiries can be directed to the corresponding author.

## Footnotes

^1^ https://www.ncbi.nlm.nih.gov/geo/

^2^ http://www.string-db.org/

^3^ https://www.networkanalyst.ca/NetworkAnalyst/

## Glossary

AD: Alzheimer’s disease
Aβ: β-amyloidprotein
AUC: area under the curve
MAPT: microtubule-associated protein-tau
PET: positron emission tomography
GEO: Gene Expression Omnibus
DEGs: Differentially Expressed Genes
WGCNA: weighted gene co-expression network analysis
PPI: protein-protein interaction
GO: Gene ontology
KEGG: Kyoto encyclopedia of genes and genomes
BP: biological process
CC: cellular component
MF: molecular function
LASSO: least absolute shrinkage and selection operator
SVM-RFE: support vector machine-recursive feature elimination
XGboost: Extreme Gradient Boosting
ROC: receiver-operating characteristic curve
DCA: clinical decision curve
MMSE: Minimum Mental State Examination
HDL: High-density lipoprotein
LDL: Low-Density Lipoprotein
